# Targeting CDK9 in Cancer: An Integrated Approach of Combining In Silico Screening with Experimental Validation for Novel Degraders

**DOI:** 10.3390/cimb46030111

**Published:** 2024-02-22

**Authors:** Mahesh Koirala, Mario DiPaola

**Affiliations:** Therabene Inc., Mansfield, MA 02048, USA; mahesh@therabene.com

**Keywords:** cancer, protein degradation, degraders, drug discovery, PROTAC, CDK9

## Abstract

The persistent threat of cancer remains a significant hurdle for global health, prompting the exploration of innovative approaches in the quest for successful therapeutic interventions. Cyclin-dependent kinase 9 (CDK9), a central player in transcription regulation and cell cycle progression, has emerged as a promising target to combat cancer. Its pivotal role in oncogenic pathways and the pressing need for novel cancer treatments has propelled CDK9 into the spotlight of drug discovery efforts. This article presents a comprehensive study that connects a multidisciplinary approach, combining computational methodologies, experimental validation, and the transformative Proteolysis-Targeting Chimera (PROTAC) technology. By uniting these diverse techniques, we aim to identify, characterize, and optimize a new class of degraders targeting CDK9. We explore these compounds for targeted protein degradation, offering a novel and potentially effective approach to cancer therapy. This cohesive strategy utilizes the combination of computational predictions and experimental insights, with the goal of advancing the development of effective anticancer therapeutics, targeting CDK9.

## 1. Introduction

Cancer is one of the most overwhelming challenges to global public health, with an ever-evolving landscape of complexities, requiring innovative and effective therapeutic solutions. Affecting people of all ages, it is the second most common cause of death globally. To meet this challenge, it is important to constantly explore different perspectives. Cyclin-dependent kinase 9 (CDK9), a key regulator of transcription and cell cycle progression, has been identified as a promising path for cancer therapy [1,2,3,4].

CDK9, a member of the cyclin-dependent kinase (CDK) family with its regulatory partner cyclin T1(CCNT1), forms the positive transcription extension factor b(P-TEFb) complex [5]. This complex co-ordinates the phosphorylation of RNA polymerase II and the liberation of paused RNA polymerase II, facilitating proficient transcriptional elongation of genes critical to cell cycle control and dodging apoptosis [6,7]. There is also evidence that CDK9-driven phosphorylation has been known to promote many cancers [8].

CDK9 drives vital oncogenic pathways, and its frequent overexpression across various cancer types emphasizes its significance as a prime target for cancer therapy [9,10]. The potential for overriding CDK9 may lead to the disruption of transcriptional programs that fuel cancer evolution, positioning it as a compelling scheme in pursuing novel anticancer therapeutics [11]. In fact, several CDK9 inhibitors have recently been designed through molecular modeling techniques and showed good in vitro antitumoral activity [12].

Traditional drug discovery methods repeatedly revolve around developing small molecules designed to inhibit the enzymatic activity of a target protein [13]. These approaches have yielded prominent successes with different kinase inhibitors, but they may not fully exploit the manifold functions of a target protein. While addressing this limitation and endeavoring for enhanced therapeutic efficacy, this article presents a comprehensive study that harmonizes computational methodologies, experimental validation, and the groundbreaking realm of Proteolysis-Targeting Chimeras (PROTACs) [14]. This approach aims to identify and exemplify novel CDK9 degraders and explore the new territory of targeted protein degradation, steering a significant shift in cancer drug discovery.

Our study, making use of computational predictions, experimental insights, and PROTAC-mediated protein degradation, seeks to bridge the considerable gap between target identification and the clinical development of effective therapies. In the upcoming sections, we will present the computational and experimental methodologies employed in our investigation, along with results, thus highlighting their synergistic potential to drive the discovery of novel CDK9-targeted cancer therapeutics.

## 2. Materials and Methods

### 2.1. Computational Methods

#### 2.1.1. Compound Selection and Analysis

Candidate inhibitors targeting CDK9 were chosen through an extensive review of chemical databases and relevant literature. A subset of these inhibitors, already present in our in-house inventory and previously subjected to testing, remains actively under consideration for ongoing research initiatives [7,15,16]. Using Openbabel [17] to expand our repertoire, a comprehensive database search was conducted using a similarity search approach. Enamine library [18], comprising a diverse array of chemical compounds, was used for this. Approximately 10,000 combinations were scrutinized, subjected to rigorous filtration, and systematically sorted to identify compounds with potential inclusion in our study. This systematic approach not only broadens the scope of our investigation but also ensures that the selected inhibitors exhibit a range of structural and chemical characteristics, enriching the diversity of our research dataset.

#### 2.1.2. Target Preparation

The structural examination of CDK9 involved utilizing tools for structural modeling, visualization, and refinement, as outlined in references [19,20,21]. To ensure a robust foundation, UniProt (P50750) [22] was employed to identify potential CDK9 structures, with a particular emphasis on selecting the most optimal structure (PDB ID: 3 BLR) [23,24]. This selection was based on criteria such as resolution, the absence of missing residues, and alignment with other available systems, ensuring a high-quality structural template for subsequent analyses [20,22].

Furthermore, an in-depth investigation was conducted using X-ray data to identify the existing ligand-binding pocket. Additionally, the exploration of potential alternative active sites was undertaken by applying CAVIAR [25]. This comprehensive structural analysis provides a reliable structural framework for CDK9 and lays the groundwork for a nuanced understanding of its ligand interactions and potential regulatory sites.

#### 2.1.3. Molecular Docking

The molecular docking of compound collections and CDK9 was executed through AutoDock-Vina [26], employing Smina and the latest version of Vina (version 1.2.5) for docking and scoring methods [27,28]. The compounds resulting from the docking process were then meticulously arranged based on their binding affinity, providing a systematic ranking of their potential interactions with CDK9. To ensure the accuracy of the docking simulations, the receptor and ligands were meticulously prepared using AutoDock tools [26]. In assessing the performance of CDK9 with its prospective novel inhibitors, a comparative analysis was conducted against other cyclin-dependent kinases (CDKs) utilizing the DUDE dataset for docking and scoring [29]. Among the identified compounds, the one with the highest binding affinity was selected for further investigation, designated as TB0016. This meticulous selection ensures that the combination chosen for in-depth study exhibits the most favorable binding characteristics with CDK9.

Moreover, in addition to TB0016, two previously identified compounds under examination by Therabene, namely TB003 and TB008, were also included in our study. All the compounds analyzed in our research are collectively referred to as TB compounds. This comprehensive approach involving diverse compounds, each selected based on their docking performance, enriches the breadth and depth of our investigation into potential inhibitors for CDK9.

#### 2.1.4. ADMET Properties

The selected compound from the docking (TB0016) and two other compounds (TB003 and TB008) were analyzed with SwissADME [30] to study the ADMET properties (absorption, distribution, metabolism, elimination, and toxicity). Integrating these properties is expected to enhance the overall understanding of the compound’s pharmacokinetic properties, aiding in the early identification and mitigation of potential safety concerns.

#### 2.1.5. Molecular Dynamics Calculations

To assess the stability of the chosen compounds identified through library screening and docking within the CDK9 complex, a comprehensive evaluation was conducted using molecular dynamics (MD) simulations facilitated by the GROMACS package version 2021.4-Ubuntu-2021.4-2 [31,32]. These simulations were carried out in a box, explicitly including water and ions, and employed the CHARMM36 (charm36Jul2022.ff) force field to represent molecular interactions accurately [33]. The ligand’s topology was meticulously prepared using the external tool CGenFF [34].

During the simulation, the system underwent position restraint, employing a 1000 kJ mol^−1^ nm^−2^ force constant to stabilize the system and prevent unrealistic structural deviations. The simulation plan included an initial energy minimization through steepest descent minimization with 10,000 steps, each with a minimization step size of 0.01. Subsequently, NVT (canonical ensemble) and NPT (isothermal–isobaric ensemble) equilibration steps were conducted, each lasting one nanosecond, with a time step of 2 femtoseconds. The final production run extended over 50 nanoseconds, providing a prolonged timeframe for observing and assessing the stability of the compounds within the CDK9 complex. Subsequently, trajectory analysis, including modules such as RMSD, RMSF, gyration, hydrogen bonds, and solvent-accessible surface area (SASA), was performed to investigate the dynamics of the simulated system. This MD simulation approach enhances our understanding of the selected compounds’ dynamic behavior and structural stability during the simulated timespan.

### 2.2. Experimental Methods

#### 2.2.1. Chemical Compounds

TB003 and TB008 are proprietary PROTAC structures; briefly, the constructs of these two molecules comprise two functional moieties, one that binds to the target protein, CDK9, and the other that binds to cereblon E3 ligase. The two moieties are linked through an alkyl chain or polyethylene glycol linker. These molecules range in size between 750 g/mole and 950 g/mole and are relatively hydrophobic. The structure of TB0016 is also proprietary; while the TB0016 degrader precursor has been synthesized and subjected to some biochemical characterization, the TB0016 degrader has yet to be synthesized.

#### 2.2.2. Biochemical Assays

##### Adapta Assay

The Adapta universal kinase assay operates through two phases: a kinase reaction phase and an ADP detection phase. During the kinase reaction phase, all components necessary for the reaction are introduced into the well, allowing incubation for 60 min. Following the response, a detection solution is applied, comprising a europium-labeled anti-ADP antibody, an Alexa Fluor™ 647 labeled ADP tracer, and EDTA to halt the kinase reaction. ADP generated by the kinase reaction displaces the Alexa Fluor 647 labeled ADP tracer from the antibody, reducing the TR-FRET signal. In the presence of a kinase inhibitor, the formation of ADP is diminished, and the ensuing interaction between the intact antibody and tracer leads to a heightened TR-FRET signal.

The assay procedure entails using a multi-well plate. It involves the following steps: initially, 100 nL of CDK inhibitors (concentrations ranging from 0.1 nM to 1 µM) in an aqueous solution with up to 1% DMSO is added to the wells. Subsequently, 2.4 µL of 30 mM HEPES (pH 7.5), 2.5 µL of a 4X ATP solution in water, and 5 µL of a 2X Kinase Mixture in 50 mM HEPES (pH 7.5), 0.01% BRIJ-35, 10 mM MgCl2, and 1 mM EGTA are introduced into each well. The plate is then shaken for 30 s and centrifuged at 1000× *g* for 1 min. The reaction mixture undergoes incubation at room temperature for 60 min. Subsequently, 5 µL of a detection mix containing 30 mM EDTA, 6 nM Eu-anti-ADP antibody, and ADP tracer is added to each well. After shaking for 30 s and centrifugation, the mixture is equilibrated at room temperature for 60 min. Finally, the assay is read on a fluorescence plate reader and the data are analyzed. This detailed assay procedure facilitates the assessment of CDK inhibitors’ inhibitory effects on kinase activity, providing a systematic approach for experimentation and ensuring the generation of reliable data for subsequent analysis.

##### Z-Lyte Assay

The Z-LYTE biochemical assay utilizes a coupled-enzyme format with a fluorescence-based approach. Its methodology relies on the differential sensitivity of phosphorylated and non-phosphorylated peptide substrates to proteolytic cleavage. The peptide substrate, labeled with fluorophores at each end, constitutes a FRET pair. In the primary reaction, the kinase transfers the gamma-phosphate of ATP to a specific tyrosine, serine, or threonine residue in the synthetic FRET-peptide. Subsequently, a site-specific protease recognizes and cleaves any non-phosphorylated FRET-peptide in the secondary response, as phosphorylation prevents peptide cleavage. Cleavage disrupts FRET between the donor (coumarin) and acceptor (fluorescein) fluorophores on the FRET-peptide, while uncleaved, phosphorylated FRET-peptide maintains the FRET signal. A ratiometric method, determining the emission ratio by calculating the ratio of coumarin emission (445 nm) to FRET (fluorescein) emission (520 nm) after exciting the donor fluorophore at 400 nm, is employed for quantifying reaction progress.

The assay procedure involves introducing inhibitory test samples (100 nL) with concentrations ranging from 0.1 nM to 1 µM in water with up to 1% DMSO onto a multi-well plate. Subsequently, 2.4 µL of kinase buffer, 5 µL of a 2X Peptide (4 µM)/Kinase (2 ng) Mixture, and 2.5 µL of 4X ATP (40µM) are successively added to each well. Following a 30 s shaking period, the reaction mixture undergoes incubation at room temperature for 60 min. Afterward, 5 µL of a proprietary Development Reagent Solution is added and the plate is shaken for another 30 s. The reaction is performed at room temperature for 60 min before being read on a fluorescence plate reader. The resulting data are then analyzed to evaluate the inhibitory effects of the test samples on kinase activity.

#### 2.2.3. CDK9 Degradation with TB003 and TB008

To demonstrate that TB003 works as a degrader of CDK9, Malme 3M cells (ATCC, HTB-64), MIA-PaCa-2 cells (ATCC, CVRM-CRL-1420), and NCI-H358 cells (ATCC, CRL-5807) were grown to confluency in 35 × 12 mm cell culture dishes using IMDM media (ATCC, Cat # 30-2005) with 20% bovine serum (3M cells, ATCC cat # 30-2020), DMEM media (ATCC, cat # 30-2002) with 10% bovine serum (MIA-PaCa), or RPMI-1640 media (Gibco, Grand Island, NY, USA, Cat # 11675) with 10% bovine serum (NCI-H358), respectively. To evaluate TB003, 3M cells were treated with and without 1 µM TB003 for 2, 4, 8, and 12 h at 37 °C. For TB008, the MIA-PaCa cells were treated for 6 h at 37 °C with varying concentrations of TB008, ranging between 0.037 μM and 3 µM. For concentration-dependent degradation, using TB003, the H358 cells were also treated for 6 h at 37 °C with TB003 at concentrations between 0.18 µM and 3 µM. After incubation, the cells from each experiment were washed with ice-cold PBS and lysed with cell lysis buffer (Cell Signaling Technology, Danvers, MA, USA, Cat # 9803S) containing 1X protease cocktail (Thermo Scientific, Rockland, IL, USA, Cat # 78442). After removing cellular and nuclear debris by centrifugation at 13,000 rpm (Eppendorf, Enfield, CT, USA) for 10 min, a 75 µL aliquot was mixed with 4X reducing Laemmli sample buffer (Boston BioProducts, Milford, MA, USA, Cat # BP-111R) and loaded onto a 4–12% Bis-Tris gel (GenScript, Piscataway, NJ, USA, Cat # M00652). The gel was run at 125 V for 60–90 min and then electro-transferred onto PVDF (Bio-Rad, Hercules, CA, USA, Cat # 162-0177). After transfer, the membrane was blocked with 5% dry-milk powder (Boston Bioproducts, Milford, MA, USA, Cat # P-1400) and developed using a combination of rabbit anti-CDK9 (Cell Signaling Technology, Cat # 2316S) and anti-myosin light-chain monoclonal antibodies (Cell Signaling Technology, Cat # 8505S), used at 1:1500 dilution and 1:5000, respectively, followed by goat anti-rabbit antibody conjugated with HRP (Cell Signaling Technology, Cat # 7074S) at 1:10,000 dilution. Bands on the membrane were visualized and imaged by chemiluminescence (Aplegen, Omega Lum G) using an ECL reagent (Thermo Scientific, Cat # A38554) [35,36].

Bands on each Western blot were digitized and quantified using densitometry software (Licor–Image Studio, Version 5.2).

#### 2.2.4. Effect of Degrader on Cell Viability

The cell lines used in this study were purchased from ATCC (https://www.atcc.org, accessed on 18 December 2023) and maintained under standard conditions at 37 °C with a 5% CO_2_ atmosphere. NCI-H-358 cells were cultivated in 1X RPMI 1640 medium, while MIA-PaCa2 cells were cultured using 1X DMEM. Both culture media were supplemented with 10% fetal bovine serum and 1X Penicillin/Streptomycin (Gibco). For culturing the Melma-3M cells, IMDM media fortified with 20% fetal bovine serum and 1X Penicillin/Streptomycin was used. To prevent overgrowth, cells were regularly split using 0.25% trypsin (Thermo-Fisher, Waltham, MA, USA) before reaching full confluence, and the culture media were refreshed every 3–4 days.

This cell culture protocol ensured appropriate conditions for assessing the degrader’s impact on cell viability. The choice of diverse cell lines and the consistent cultivation practices contributed to reliable results and relevance in elucidating the degrader’s effects on cellular viability.

Briefly, for cell viability experiments, after splitting, cells from each respective cell line were plated onto 96-well plates (MSP, Cat # CT-229185) at a density of about 1 × 10^4^ cells/mL and 200 µL per well using the appropriate cell culture media after about 24 h at 37 °C, the cell media in each 96-well plate were replaced with media containing the various test compounds at varying concentrations. The culture plates were returned to the incubator and incubated at 37 °C for another 48 to 72 h. After this last incubation, the media from each plate were discarded, and each plate was washed 2X with cold (5 °C) PBS (Gibco, Grand Island, NY, USA, Cat # 70011). After the last wash, a 100 µL volume of 1:1 (V:V) mix of PBS and cell-titer-glo (Promega, Chuo City, Japan, Cat # G8461) was added to each well on each culture plate. Each plate was gently mixed for ca. 10 min at room temperature and then transferred to a plate reader (Bio-Tek, Synergy HTX, Winooski, VT, USA, multimode reader) for chemiluminescence measurement as a proportional indicator of cell viability.

#### 2.2.5. Graphical Presentations and Statistical Analysis

Statistical analyses and graphical presentations were performed using Prism (GraphPad, version 10.1.2).

## 3. Results

### 3.1. Computational Results

#### 3.1.1. Docking Study

**Figure 1:**
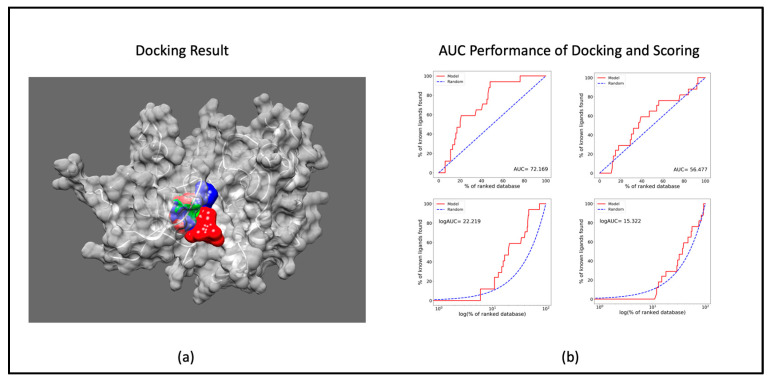
Docking and scoring results: (**a**) docked pose of TB compounds to CDK9 (PDB ID 3BLR) [23,24] (red: TB003; blue: TB008; and green: TB0016); (**b**) AUC values of docking and scoring CDK9 (**left**) and CDK2 (**right**).

The outcomes of the molecular docking analysis involving CDK9 and Therabene’s proprietary compounds (TB003, TB008, and TB0016) (these are proprietary chemical entities and structural details are not revealed due to ongoing patenting activities) are documented in Table 1. The docking results elucidate the optimal binding sites of the ligands with the receptor, highlighting the species of the most stable binding determined by the binding affinity (Figure 1a). Notably, the docking performance of CDK9 surpasses that of other cyclin-dependent kinases (CDKs), as evidenced by a superior Area Under the Curve (AUC) value. Examining the AUC performance in Figure 1b reveals that CDK9 outperforms CDK2. This outstanding AUC performance of CDK9 can be anticipated to extend to other CDKs. The data thus underscore the efficacy of CDK9 in ligand binding, suggesting a notable advantage over its CDK counterparts based on our comprehensive analysis of molecular docking results.

#### 3.1.2. ADMET Analysis

The physicochemical properties of the compounds and their PROTAC, which serve as the descriptors to evaluate their bioactivity, are shown in Table 2. TB003 exhibits high lipophilicity with a LogP value 5.09, signifying a strong affinity for lipid environments. However, its low absorption and poor solubility, with a LogS of −6.29, suggest potential challenges in bioavailability. In contrast, TB008 demonstrates moderate lipophilicity (LogP 2.75), coupled with high absorption and good solubility (LogS −3.83). TB0016 shares similar attributes with TB008, displaying moderate lipophilicity (LogP 2.55), high absorption, and moderately soluble characteristics (LogS −4.28). The PROTAC versions of TB003 and TB008 also exhibit high lipophilicity but their low absorption and moderately to poorly soluble nature could pose formulation challenges in drug development. These compound profiles underscore the importance of balancing lipophilicity, absorption, and solubility for optimal drug design and delivery. The PROTAC version of TB0016 is currently being made.

In summary, all drugs, including precursors and PROTACs, display LogP values that fall well within the desirable range for therapeutic compounds. As expected, PROTAC molecules, however, are less soluble than the precursor compounds and, also, because of size, the PROTACs are not as well absorbed.

#### 3.1.3. MD Simulations

The molecular dynamics (MD) simulations conducted for each complex yielded valuable insights into the intricate molecular interactions between the target and our selected compounds. This comprehensive analysis delves into the nuances of the complexes’ stability and dynamics under scrutiny. In addition to the simulations of CDK9 alone, the complexes subjected to MD simulations include CDK9-TB003, CDK9-TB008, and CDK9-TB0016, allowing for a detailed exploration of their behavior at the molecular level. These simulations show how these complexes evolve, unraveling essential information about their structural stability and dynamic behavior.

##### RMSD Analysis

To investigate the structural conformations of CDK9, CDK9-TB003, CDK9-TB008, and CDK9-TB0016 at the molecular level, the analysis focused on calculating Root Mean Square Deviations (RMSD) values. The mean RMSD values for CDK9, CDK9-TB003, CDK9-TB008, and CDK9-TB0016 were quantified as 0.29 nm, 0.30 nm, 0.28 nm, and 0.27 nm, respectively. These outcomes suggest minimal variations in the structural configurations of the complexes following binding events.

**Figure 2:**
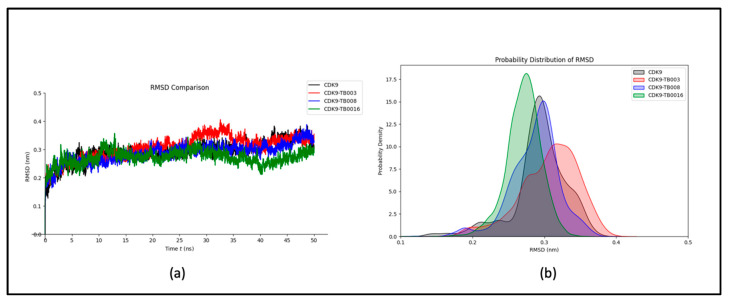
RMSD analysis: (**a**) RMSD plot of CDK9 and CDK9-TB compounds; (**b**) probability distribution.

The RMSD plot, depicted in Figure 2a, visually represents the sustained stability observed throughout the simulation. The plot demonstrates a consistent trajectory, indicating that the complexes maintain high structural integrity over the simulation period. This implies that the binding of the ligands to CDK9 does not induce significant structural deviations.

Furthermore, the probability distribution analysis, as illustrated in Figure 2b, corroborates the stability of the complexes by revealing a lack of significant shifts. The probability distribution graph provides additional evidence supporting the notion that the structural integrity of each complex remains reasonably stable throughout the simulation, further substantiating the robustness of the molecular systems under investigation.

##### RMSF Analysis

**Figure 3:**
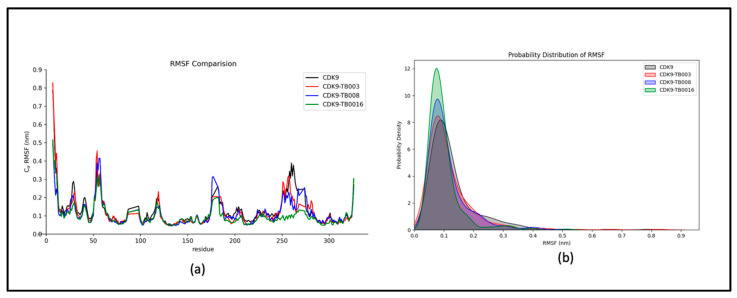
RMSF analysis: (**a**) RMSF fluctuations of CDK9 and CDK9-TB compounds; (**b**) probability distribution.

Examining dynamic characteristics and flexibility within the complex employs Root Mean Square Fluctuation (RMSF) analysis, as shown in Figure 3a. The results reveal that residual fluctuations consistently display stability and remain minimized throughout the simulation duration. Notably, subtle changes are observable within the residues ranging from 250 to 300, positioned distally from the binding site at the protein’s terminus.

The probability distribution plot of RMSF, as illustrated in Figure 3b, reinforces the system’s stability by indicating an absence of significant shifts. This graphical representation further underscores the system’s overall strength, emphasizing the minimal impact of fluctuations on the dynamic behavior of the complex.

##### Radius of Gyration Analysis

**Figure 4:**
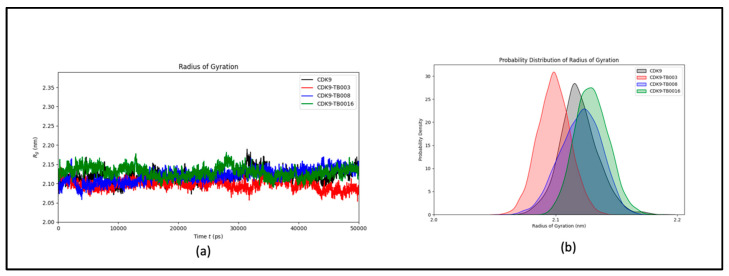
The radius of gyration of CDK9 and CDK9-TB compounds: (**a**) plot of R_g_ over time; (**b**) probability distribution of R_g_.

The examination considered the structure’s intricacy and overall configuration before and after ligand binding, utilizing the gyration radius (R_g_) metric throughout the simulation. The findings indicate that the protein’s stability remained predominantly unaltered by the binding event, as evidenced by the consistent R_g_ values observed throughout the simulation duration, as illustrated in Figure 4a. This sustained stability highlights the robustness of the protein structure, implying that the ligand binding did not elicit notable structural disruptions. The probability distribution plot (Figure 4b) further supports the comparable stability of the system, revealing no significant shifts in R_g_ values.

##### Analysis of Solvent-Accessible Surface Area (SASA)

**Figure 5:**
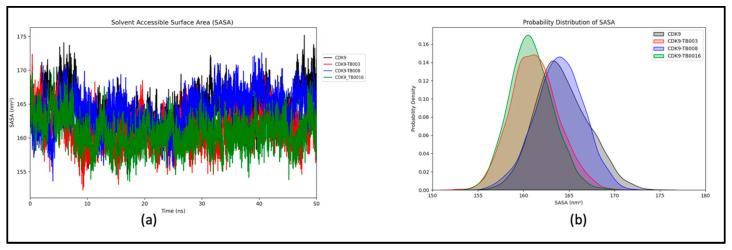
Solvent-accessible surface area (SASA) analysis: (**a**) SASA of CDK9 and CDK9-TB compounds; (**b**) probability distribution of SASA.

We conducted an in-depth examination of the protein’s interaction with its surrounding solvent by computing the solvent-accessible surface area (SASA) for CDK9 and CDK9-TB complexes. The calculated average SASA values for CDK9, CDK9-TB003, CDK9-TB008, and CDK9-TB0016 were determined to be 163.98 nm^2^, 161.00 nm^2^, 163.68 nm^2^, and 160.62 nm^2^, respectively, as depicted in Figure 5a. This analysis offers valuable insights into the extent of protein exposure to the solvent in each complex.

Moreover, the probability distribution plot (Figure 5b) further reinforces that the systems, including CDK9, CDK9-TB003, CDK9-TB008, and CDK9-TB0016, remained stable throughout the simulation. This stability is a critical factor in understanding the structural integrity and behavior of these complexes, providing additional confidence in the reliability of the obtained results.

##### Hydrogen Bonds Dynamics

**Figure 6:**
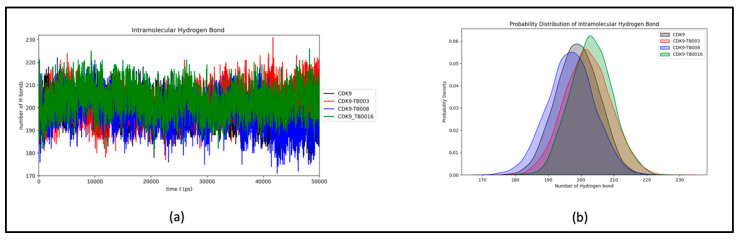
Intramolecular hydrogen bond dynamics: (**a**) number of hydrogen bonds over time; (**b**) probability distribution of intramolecular hydrogen bond.

Preserving hydrogen bond stability during simulations is imperative for upholding the structural conformation of the protein and its complexes. In the context of CDK9 and its interactions with TB003, TB008, and TB0016, the mean count of intramolecular hydrogen bonds was consistently recorded at 198, 202, 197, and 203, respectively. This unwavering count emphasizes the stability of hydrogen bonding interactions between CDK9 and the CDK9-TB compounds throughout the simulation, as portrayed in Figure 6a. Notably, the slight elevation in the number of hydrogen bonds can be ascribed to the increased complexity inherent in the CDK9-TB complexes. The probability distribution plot of the number of hydrogen bonds further substantiates the system’s stability during the simulation, as depicted in Figure 6b.

**Figure 7:**
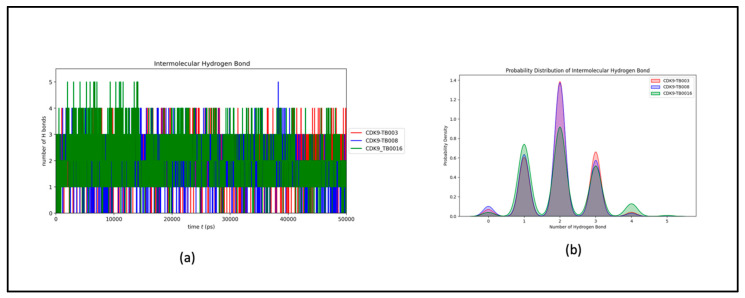
Intermolecular hydrogen bond dynamics: (**a**) number of hydrogen bonds between CDK9 and TB compounds; (**b**) probability distribution of intermolecular hydrogen bond.

The intermolecular hydrogen bonds observed between CDK9 and TB compounds exhibit variability, extending up to five, yet with a noticeable consistency within the one to three bond range. This consistency reflects minimal fluctuations throughout the simulation, as illustrated in Figure 7a and further elucidated in its distribution plot, Figure 7b. The findings affirm that the ligands consistently maintained their initial binding positions from the docking phase throughout the simulation, corroborating the sustained stability of the hydrogen bonding interactions within the CDK9-TB complexes.

### 3.2. Experimental Results

#### 3.2.1. Assay

The table presented below illustrates the notable effectiveness of both TB003 and TB008 in inhibiting the kinase activity of the CDK9/Cyclin T complex. Conversely, TB0016 exhibited no discernible inhibitory properties (Table 3).

Notably, TB003 and TB008 emerge as potent CDK9 degraders, with IC50 values of 5 nM and 3.5 nM, respectively. In contrast, TB0016, identified as a CDK9 ligand, exhibits no significant potency with an IC50 greater than 1 µM. Further emphasizing the selectivity of TB003 and TB008 for CDK9, the fold selectivity values against other CDK family members, such as CDK7, CDK5, CDK4, CDK2, and CDK1, are provided. These compounds, suitable for administration via IP and potentially oral routes, present promising prospects for targeted therapeutic interventions.

#### 3.2.2. CDK9 Degradation by Western Blot

**Figure 8:**
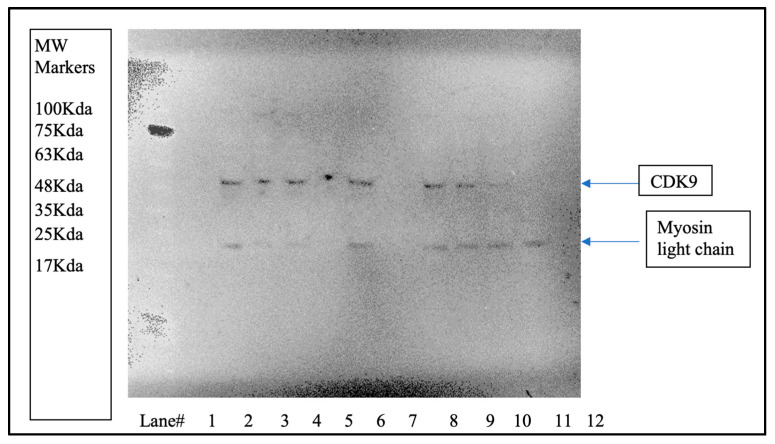
Western blot of 3M cells treated with TB003: approximately a 30 µL volume of each lysate was loaded into the gel in the following order: MW markers lane 1, control lane 3, 2 h control lane 4, 4 h control lane 5, 12 h control lane 7, 2 h treatments lane 9, 4 h treatment lane 10, 8 h treatment lane 11, and 12 h treatment lane 12.

**Figure 9:**
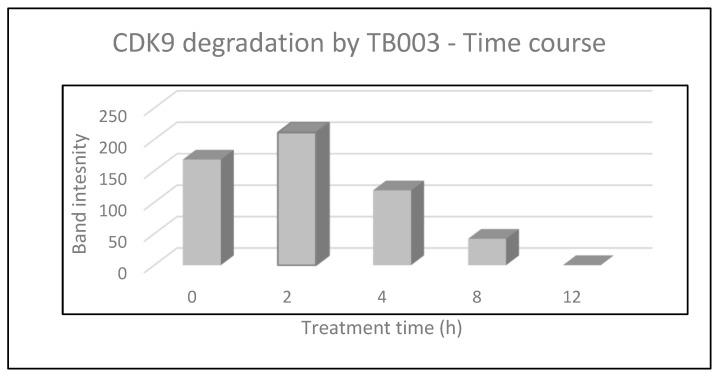
Densitometric analysis (*n* = 1) of the Western blot image shown in Figure 8.

To demonstrate that TB003 degrades CDK9 selectively, the Malme 3M cell line was grown to confluency in 35 mm culture dishes and then treated with TB003 at 1 µM for varying periods. The cell lysates from each time point, containing approximately 1–2 mg/mL of protein, were then analyzed by Western blot. An image of the Western blot is shown in Figure 8 and the corresponding densitometry is in Figure 9.

To mitigate variations in protein load, the Western blot was also stained for the light chain of myosin as a control. Notably, the myosin light chain appears consistently comparable across the lanes. However, the CDK9 band exhibits diminishing intensity with increasing treatment time, as shown by the densitometric analysis displayed in Figure 9. CDK9 band intensity in each lane was corrected for potential protein load variability using the quantified intensity of the myosin light-chain band for each lane, according to the following equation:

[Normalized CDK9 intensity] = {[myosin]_highest intensity_/[myosin intensity]} × [CDK9 intensity]

These results demonstrate that, as cells are exposed to the degrader for longer durations, there is a proportional decrease in the intensity of the CDK9 band, providing compelling evidence for the specific degradation of CDK9 induced by TB003. The dynamic changes in CDK9 intensity, as evidenced by the Western blot, reinforce the temporal relationship between TB003 treatment and the degradation of the target protein.

**Figure 10:**
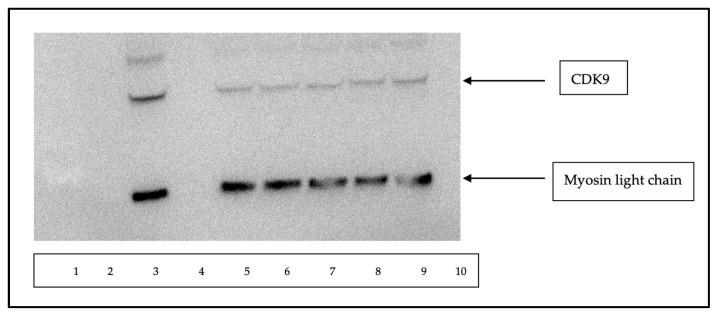
Western blot of NCI-H358 cells treated with varying concentrations of TB003 for 6 h: approximately a 25 µL volume of each lysate was loaded into the gel in the following order: lane 1, MW markers (not visible); lane 2, empty; lane 3, control; lane 4, empty; lane 5, 3 μM; lane 6, 1.5 μM; lane 7, 0.75 μM; lane 8, 0.375 µM; lane 9, 0.188 µM; and lane 10, empty.

**Figure 11:**
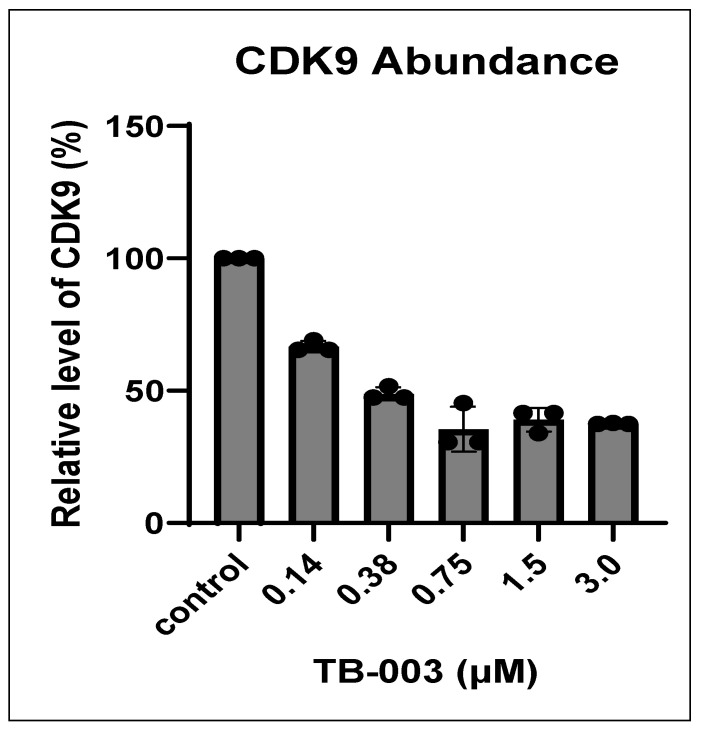
Graphical display of Western blot densitometric data (*n* = 3) from H358 cells treated with varying concentrations of TB003.

To further demonstrate the effectiveness of TB003 in degrading CDK9, NCI-H358 cells were subjected to a 6 h treatment with TB003 at concentrations ranging from 0.18 µM up to 3 µM. Figure 10 displays a representative image of the Western blots obtained from this specific set of experiments. Figure 11 includes the densitometric analysis of the Western blot banding pattern derived from three separate repeats. The obtained data show a clear and distinct reduction in CDK9 protein levels following TB003 treatment, and this reduction was found to be concentration-dependent, reaching a plateau at approximately 1 µM.

**Figure 12:**
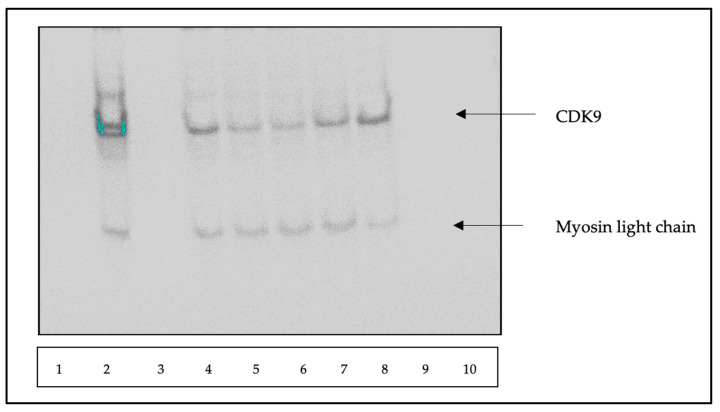
Western blot of MIA-PaCa 2 cells treated with TB008: approximately a 25 µL volume of each lysate was loaded into the gel in the following order: MW markers lane 1 (not visible), control lane 2, 3 µM lane 4, 1 μM lane 5, 0.333 µM lane 6, 0.111 μM lane 7, and 0.037 µM lane 8. Lanes 9 and 10 are empty.

**Figure 13:**
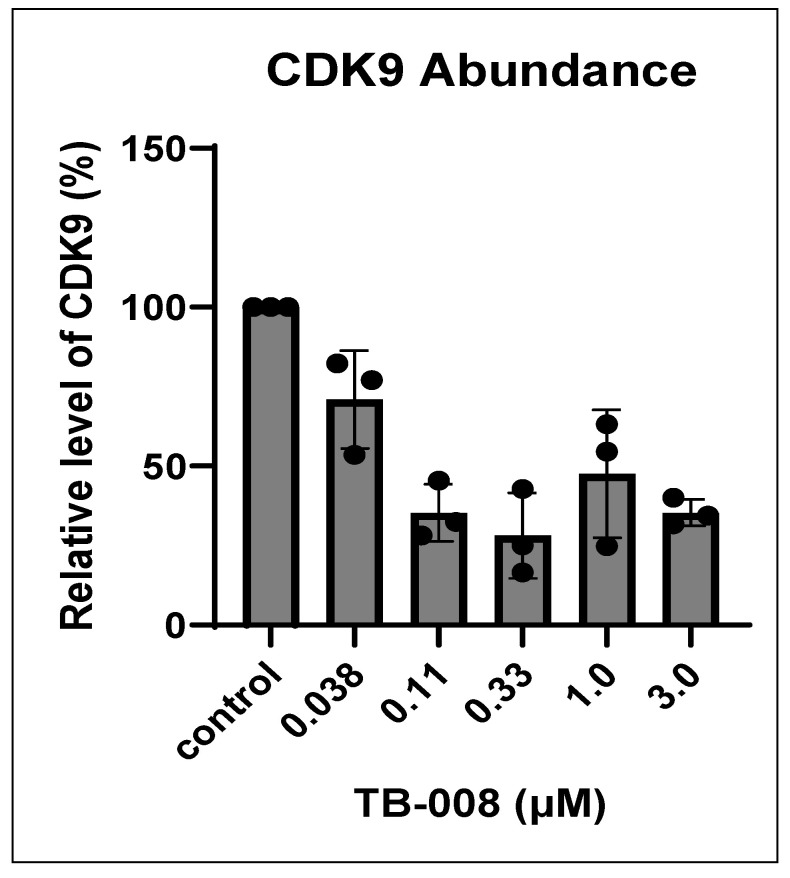
Graphical display of Western blot densitometric data (*n* = 3) from Mia-PaCa-2 cells treated with varying concentrations of TB008.

In a separate experiment, MIA-PaCa 2 cells were treated also for 6 h with varying concentrations of TB008, ranging between 0.037 µM and 3 μM. The cells were then harvested, lysed, and analyzed by Western blot. Results from this study are shown in Figure 12 and Figure 13, western blot image and densitometry, respectively.

As seen with TB003, TB008, when used on MIA-PaCa 2 cells, was quite effective at triggering CDK9 degradation in a concentration-dependent manner.

#### 3.2.3. Cell Viability

**Figure 14:**
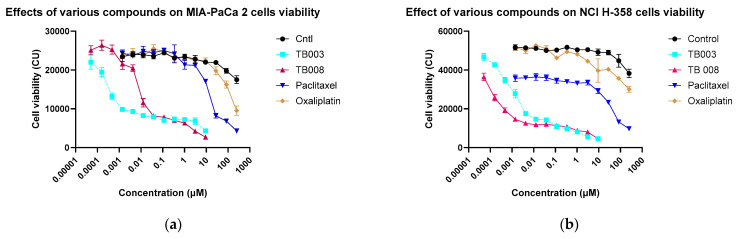
Cell viability results: (**a**) MIA-PaCa-2; (**b**) NCI H-358.

TB003 and TB008 have also been evaluated for cell growth inhibition and killing using two cell lines, NCI-H358, a non-small cell lung carcinoma-derived cell line, and MIA-PaCa-2, a pancreatic carcinoma-derived cell line. For this study, both drugs were added to cells at an initial concentration of 10 µM, then serially diluted (1:3, V:V)) down to 0.2 nM. For comparison purposes, two commonly used chemotherapeutic agents, oxaliplatin and paclitaxel, were also added to the test plates, both at an initial concentration of 250 µM and then diluted serially (1:3, V:V). Results from triplicate measurements are presented in Figure 14.

In the context of antiproliferative efficacy and cell-killing potential, both TB003 and TB008 exhibited notable effectiveness when tested against the MIA-PaCa-2 and NCI H-358 cell lines. A direct comparison with standard chemotherapeutic agents, such as paclitaxel and oxaliplatin, revealed that TB003 and TB008, with EC_50_ values in the low nanomolar range, surpassed the potency of these reference compounds (Table 4). Notably, both degraders demonstrated robust antiproliferative properties but TB008 outperformed in cell-killing efficacy, as evidenced by a cell viability signal approaching zero at elevated drug concentrations. These findings underscore the potential of TB003 and TB008 as highly effective agents in impeding cell growth and inducing cell death, positioning them as promising candidates for further therapeutic development against pancreatic cancer, as well as non-small cell lung carcinoma.

Statistical analysis of the cell viability data by multiple t-test is provided in Table 5 below.

Both TB003 and TB008 displayed strong statistical significance compared to control across all concentrations. Paclitaxel was quite effective against H358 cells across all concentrations but less effective against MIA-PaCa 2 cells, while oxaliplatin was the least effective out of the four tested compounds.

## 4. Discussions

The comprehensive approach presented in this study combines computational methodologies, experimental validation, and PROTAC technology to identify, characterize, and optimize a new class of CDK9 degraders for cancer therapy. The study begins by emphasizing the pivotal role of CDK9 in transcription regulation and cell cycle progression, positioning it as a promising target for cancer treatment. Integrating computational predictions and experimental insights seeks to bridge the gap between target identification and the clinical development of effective therapies.

The computational results, encompassing molecular docking, ADMET analysis, and MD simulations, provide valuable insights into the binding affinities, physicochemical properties, and dynamic behavior of the CDK9-TB compounds. Notably, CDK9 exhibits superior docking performance compared to other CDKs, emphasizing its efficacy in ligand binding. The MD simulations reveal the stability of the complexes, with minimal structural variations and consistent interactions between the ligands and CDK9. ADMET analysis sheds light on the pharmacological behavior of the compounds, emphasizing the importance of balancing lipophilicity, absorption, and solubility for optimal drug design.

Experimental results further validate the efficacy of TB003 and TB008 as potent CDK9 degraders, with IC_50_ values in the low nanomolar range and strong selectivity for CDK9 relative to other CDK family members. Western blot analysis demonstrates specific degradation of CDK9 induced by both TB003 and TB008, reinforcing their potential as targeted therapeutic agents. Cell viability assays highlight the antiproliferative and cell-killing efficacy of TB003 and TB008, surpassing the potency of standard chemotherapeutic agents.

In contrast to the consistency of the computational findings among the three molecules, TB0016 does not exhibit strong inhibitory effects in the CDK9 kinase assay. Therefore, a thorough examination of TB0016 is essential for a more comprehensive investigation, including assessments such as binding activity, cell viability, Western blot analysis, and exploring alternative binding interactions with CDK9. This disparity underscores the need for careful consideration and a more profound analysis when interpreting computational and molecular docking results.

In light of the in vitro results seen with TB003 and TB008 and published data showing evidence for significant overexpression of CDK9 in various cancer types, including leukemia, cervical cancer, triple-negative breast (TNB) cancer, melanoma, lung cancer, glioblastoma, endometrial cancer, and pancreatic cancer [37,38], along with the finding that selective CDK9 inhibition leads to downmodulation of c-Myc and induction of apoptosis in B-cell lymphoma cells [39], there is compelling interest in pursuing the development of these two degraders for the treatment of triple-negative breast cancer, as well as pancreatic cancer and non-small cell lung carcinoma. Indeed, in a recently conducted in vivo study using a TNB cancer cells xenograft mouse model (CDX model), TB003 was shown to significantly inhibit cancer growth during the study.

## 5. Conclusions

The integrated approach, as presented in this study, allows for the successful identification and characterization of a novel class of CDK9 degraders with promising anticancer properties. The study combines computational and experimental techniques, providing a thorough understanding of the molecular interactions, pharmacological behavior, and therapeutic potential of the CDK9-TB compounds. TB003 and TB008 emerge as promising candidates, demonstrating not only potent CDK9 inhibition but also effective induction of targeted protein degradation and robust antiproliferative effects. The findings underscore the potential of these compounds as lead candidates for further therapeutic development in pursuing innovative and effective cancer treatments, targeting CDK9. The positive computational results for TB0016 necessitate a deeper exploration of the biochemical and biological properties of TB0016 in its interactions with CDK9 and the development of its corresponding PROTAC structure. This study contributes to the evolving landscape of cancer drug discovery by presenting a holistic and multidisciplinary approach to identifying and optimizing novel therapeutic agents.

## Figures and Tables

**Table 1:** Ligands and CDK9 kinase binding affinity with different docking methods ^1^.

Ligand Name	Binding Affinity (kcal/mol)	Docking Method
TB003	−7.8	Vina
TB003	−8.4	Smina
TB008	−9.6	Vina
TB008	−9.8	Smina
TB0016	−9.7	Vina
TB0016	−8.8	Smina

^1^ These are proprietary chemical entities and structural details are not revealed due to ongoing patenting activities.

**Table 2:** Evaluation of pharmacological behavior of compounds.

Compound	Lipophilicity (LogP)	Absorption	Solubility (LogS)
TB003	5.09 (High)	Low	−6.29 (Poorly Soluble)
TB008	2.75 (Moderate)	High	−3.83 (Soluble)
TB0016	2.55 (Moderate)	High	−4.28 (Moderately Soluble)
TB003 PROTAC	4.28 (High)	Low	−6.65 (Poorly Soluble)
TB008 PROTAC	3.19 (High)	Low	−5.59 (Moderately Soluble)

**Table 3:** Summary of CDK9 kinase inhibition results ^1^.

Compound		TB003	TB0016	TB008
Modality		CDK9 degrader	CDK9 ligand	CDK9 degrader
Potency (IC50)		5 nM	>1 µM	3.5 nM
Fold selectivity CDK9 vs. other CDK family members	CDK7	>200	>1 µM	>500
	CDK5	NT	>1 µM	>500
	CDK4	NT	>1 µM	>500
	CDK2	>200	>1 µM	>500
	CDK1	>200	>1 µM	>500
Route of administration		IP/Oral	IP/Oral	IP/Oral

^1^ This study was conducted at Thermo-Fisher by the SelectScreen screening and profiling services division.

**Table 4:** Estimated EC50 values.

Chemical Entity	Cell Line	
	NIH H-358	MIA-PaCa 2
TB003	20 nM	0.8 nM
TB008	0.3 nM	9 nM
Paclitaxel	75 µM	10 µM
Oxaliplatin	ND	100 µM

ND: not determinable.

**Table 5:** Statistical analysis of viability data.

Cell Type		Test Compound			
		TB003	TB008	Paclitaxel	Oxaliplatin
H358	Control	<0.001	<0.001	<0.001	<0.1; No SS at concentrations < 0.11 µM
MIA-PaCa	Control	<0.001	<0.001	<0.067; No SS at concentrations < 1 µM	<0.026; No SS at concentrations < 28 µM

SS: statistical significance.

## Data Availability

Data can be provided by directly contacting the authors.
